# 3D pelvic cadaver model: a novel approach to surgical training for penile implant surgery

**DOI:** 10.1038/s41443-019-0211-2

**Published:** 2019-10-24

**Authors:** Koenraad van Renterghem, Ahmed Ghazi

**Affiliations:** 1 Department of Urology, Jessa Hospital, Hasselt, Belgium; 2 Faculty of medicine, Hasselt University, Hasselt, Belgium; 3 University Hospitals Leuven, Leuven, Belgium; 4 Department of Urology, University of Rochester, Rochester, NY USA; 5 Simulation Innovation Laboratory, Rochester, NY USA

**Keywords:** Translational research, Anatomy

A well-known problem in surgical training is the fact that many young surgeons have insufficient exposure to practical surgical training once they start their own career operating their own patients. The Halsted principle of “see one, do one, teach one” has run its course and is at best outdated given the recent financial, medico-legal, and ethical considerations of intraoperative training. Furthermore, the implementation of shorter residency training programs and resident work-hour restrictions has resulted in several training programs incorporating simulation into their training curriculum to supplement the hands-on experience gained in the operating room. Therefore, safe and effective training modalities that fill the created gap are required. To meet these needs, we developed a training platform that enables us to offer alternative possibilities for the training of young residents and to provide opportunities for practicing surgeons to pick up new techniques or to become more experienced in certain techniques and specific surgical procedures. This educational strategy consists of sequential levels of training. Primarily, a theoretical level using dedicated handbooks is to be followed by an interactive online library where surgical procedures can be learned using interactive videos displaying the surgery from different angles and including anatomical drawings. The videos can be mastered using a touch screen from a tablet, smart phone, laptop, desktop, etc. The third level in this training curriculum utilizes three-dimensional (3D) pelvic cadaver models. Once surgical techniques are adequately acquired using these 3D models, trainees are allowed to continue training in cadaver settings. This enables us to use these precious cadaver models much more efficient. Given the fact that cadaver models are much more expensive than 3D models, besides the ethical aspects of using human bodies, including those 3D models is an important step forward in surgical training. Last but not least, training and surgical education takes place in centres of excellence under direct supervision of high-volume dedicated surgeons. Once trainees have completed this training module they can become safety certified to offer their patients these types of interventions [1–6].

**Fig. 1:**
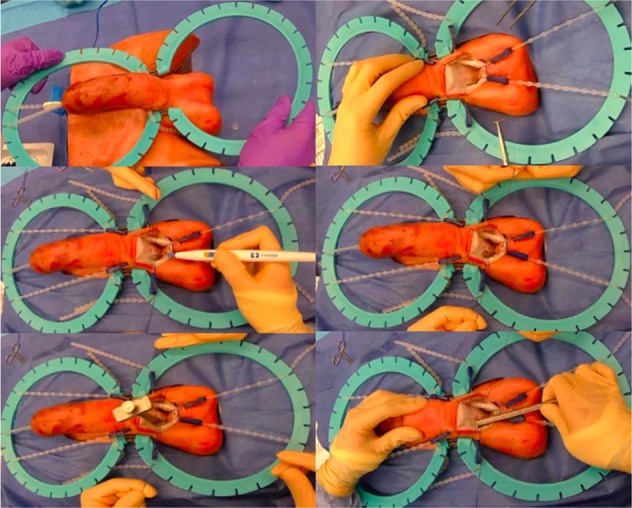
▓

Cadaveric models remain the gold standard for realistic procedural instruction but their high cost, regulated availability, risks of transferable diseases, and potential ethical concerns limit their widespread use. Nevertheless, they restrict operative practice with specific pathology or anatomic variability that is required to achieve proficiency in advanced surgical skills. Researchers at the Simulation Innovation Lab (University of Rochester Medical Centre, Rochester, New York) have created a high fidelity, nonbiohazardous, and cost-effective penile model to simulate penile prosthetics surgery. The model is intended to help supplement resident, fellow, and low-volume penile prosthesis surgeon training to increase procedural knowledge and confidence with placement of a penile prosthesis. The penile model is composed of a synthetic hydrogel tissue that can mimic the biomechanical properties of human tissue. The model is made using polyvinyl alcohol (PVA) powder that is dissolved to create a viscous gel. To retain the geometry of various male pelvic structures PVA gel is molded in plastic casts created utilizing 3D printing. The casts are designed from a Computer-Aided Design model (Fig. 1) representing the geometry of the target anatomy. To replicate the texture of various tissue components, the PVA is solidified through successive freeze–thaw cycles that result in different densities of the PVA. This process is used to create each anatomic male genital structure including: corporal bodies, urethra, spermatic cord, scrotum, testicles, inguinal canal, and iliac vessels. These structures are layered in an anatomical fashion around a 3D-printed pubic bone. Simulated fascia and ligaments are incorporated to combine the components and a skin layer is added to the outside.

**Fig. 2:**
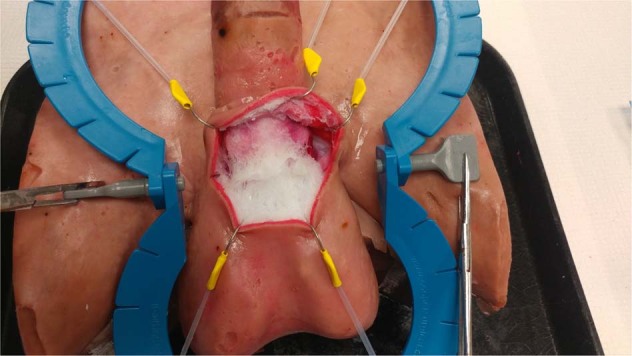
▓

In our curriculum, this 3D model would precede the wet lab as dry lab training. This 3D model is realistic in terms of appearance and texture. More importantly the model replicates anatomical structures: skin, connective tissue, urethra (that can be catheterized), tunica albuginea (where stay sutures can be placed), spongious tissue (that can be dilated), crural bodies that are attached to the ramus inferior of the pubic bone, the tubercle at the level of the symphysis as well as the inguinal ligament, the external orifice of the inguinal canal with the spermatic cord, the external oblique muscle fascia, the iliac vessels (that are hollow and can be filled with colored liquid), retzius space, etc. Therefore, the platform has the ability to replicate all appropriate steps of inflatable penile prosthetic surgery: corporal exposure, corporal dilation, appropriate measurement, corporal closure, reservoir placement, pump insertion, prosthesis connections, and closure (Fig. 2). The platform is also able to demonstrate important surgical errors including: perforation of the corporal bodies, injury to iliac vessels, and injury to urethra. The development of these 3D models is an ongoing process since improvements can be incorporated anytime [6, 7].

The use of 3D printed platforms models in surgical training is a recent development that has many appearances. The simplest of these are platforms used to train in laparoscopy where the model consists of a simple set up as a pelvic trainer with simple objects inside. More advanced models have been developed for training in adrenal cancer, renal cancer, urethro-vesical anastomosis [8], prostate cancer, pelvicalyceal junction procedures, and endourethral surgery. However, none of these can replicate the mechanical properties of living tissue. We believe this innovation presents a leading edge in surgical training that is more sophisticated. This model makes it possible to mimic all critical steps of penile implant surgery, which is considered a very technical demanding procedure. With simple modifications this model can be adapted for other reconstructive procedures including; peyronies disease, such as plication, grafting, training for male sling procedures, artificial urinary sphincter, etc. Changing this model into a female pelvic 3D model enables us to start training in female urology procedures, such as pelvic organ prolapse surgery.

The 3D virtual design also has the potentially to be useful in preoperative patient counseling [9], planning of surgery besides the educational purposes [10]. Furthermore, these training tools will result in better surgical performance, which will ultimately result in improved patient outcomes. Moreover, incorporating these 3D models into a training curriculum as explained above, the training of residents, and inexperienced surgeons can and will be improved. By doing this we have only winners: in the first place our patients, furthermore the surgeons who will be much more confident offering those surgical procedures to their patients resulting in less complications and readmissions to the hospital, etc [11, 12]. This also implicates that hospitals will benefit by having a better reputation, last but not least, we will benefit as a society by organizing our medical care more efficiently with cost reduction as a logic consequence.
